# The role of exercise testing in predicting successful ambulation with a lower extremity prosthesis: a systematic literature review and clinical practice guideline

**DOI:** 10.1186/s12984-018-0401-z

**Published:** 2018-09-05

**Authors:** Tyler D. Klenow, Larry J. Mengelkoch, Phillip M. Stevens, Chris A. Ràbago, Owen T. Hill, Gail A. Latlief, Rodrigo Ruiz-Gamboa, M. Jason Highsmith

**Affiliations:** 1 Orthotic & Prosthetic Centers, Inc, 3005 Caring Way, Suite 3, Port Charlotte, FL 33952 USA; 2 University of St. Augustine for Health Sciences, St. Augustine, FL USA; 3 Hanger Clinic, Salt Lake City, UT USA; 4 University of Utah School of Medicine, Physical Medicine and Rehabilitation, Salt Lake City, UT USA; 5 Center for the Intrepid, Department of Orthopaedics and Rehabilitation, Brooke Army Medical Center, Fort Sam Houston, TX USA; 6 Extremity Trauma & Amputation Center of Excellence (EACE), San Antonio Medical Center, Fort Sam Houston, TX USA; 7 Department of Veterans Affairs, Veterans Health Administration, Regional Amputation Center, James A. Haley Veterans Hospital, Tampa, FL USA; 8 Lee Physician Group, Department of Vascular Surgery, Fort Myers, FL USA; 9 Extremity Trauma & Amputation Center of Excellence (EACE), James A. Haley Veterans Hospital, Tampa, FL USA; 10 University of South Florida. Morsani College of Medicine, School of Physical Therapy & Rehabilitation Sciences, Tampa, FL USA; 11 Army Reserves. 319th Minimal Care Detachment, Pinellas Park, FL USA

**Keywords:** Aerobic capacity, Amputee, Artificial limb, Ergometry, Limb loss, Rehabilitation, Work load

## Abstract

**Background:**

Growing discontent with the k-level system for functional classification of patients with limb loss and movement of healthcare toward evidence-based practice has resulted in the need for alternative forms of functional classification and development of clinical practice guidelines to improve access to quality prosthetic interventions. The purpose of this project was to develop and present a clinical practice recommendation for exercise testing in prosthetic patient care based on the results and synthesis of a systematic literature review.

**Methods:**

Database searches of PubMed, Google Scholar, Web of Science, and Cochrane were conducted and articles reviewed. Of the potential 1386 articles 10 met the criteria for inclusion. These articles were assessed using the critical appraisal tool of the United Kingdom National Service Framework for Long-Term Conditions. Of the 10 included articles eight were of high, one of medium, and one of low, quality. Data from these articles were synthesized into 6 empirical evidence statements, all qualifying for research grade A. These statements were used to develop the proposed clinical practice guideline.

**Results:**

While the results of this systematic review were not able to support the direct connection between cardiorespiratory performance and K-levels, the literature did support the ability of exercise testing results to predict successful prosthetic ambulation in some demographics. Both continuous maximum-intensity single lower extremity ergometer propelled by a sound limb and intermittent submaximal upper extremity ergometer protocols were found to be viable evaluation tools of cardiorespiratory fitness and function in the target population.

**Conclusion:**

The ability to sustain an exercise intensity of ≥50% of a predicted VO_2max_ value in single leg cycle ergometry testing and achievement of a sustained workload of 30 W in upper extremity ergometry testing were found to be the strongest correlates to successful ambulation with a prosthesis. VO_2_ values were found to increase in amputee subjects following a 6-week exercise program. These synthesized results of the systematic literature review regarding exercise testing in patients with loss of a lower extremity were used to develop and a present a clinical treatment pathway.

**Electronic supplementary material:**

The online version of this article (10.1186/s12984-018-0401-z) contains supplementary material, which is available to authorized users.

## Background

Candidacy for lower limb prostheses and especially access to advanced prosthetic technologies are the subject of increased scrutiny today. As healthcare costs continue to rise across the United States, regulatory positions have sought to establish policies that increase the collective confidence that the provision of a lower limb prosthesis will represent a sound investment in the health and well-being of the individual. Medicare’s current Local Coverage Determination (LCD) indicates that a determination of the medical necessity is based on the beneficiary’s potential functional abilities, which are subsequently based on such factors as the beneficiaries’ current condition and the nature of any associated medical problems [1]. In a 2011 “Dear Physician” letter, Medicare Contractors provided additional guidance on the desired contents of the medical record (Additional file 1). Stipulating that “physicians should tailor their history and examination to the individual patient’s condition,” the letter suggests that evaluation should take into account “past medical history,” “symptoms limiting ambulation,” and “other comorbidities impacting the use of a new prosthesis.” Within this context, “Cardiopulmonary examination” is also included.

While the need for an exhaustive, objective cardiopulmonary evaluation has been assigned to the discretion of the referring physician, there are instances when past medical history, symptoms and other comorbidities may suggest the need for an objective assessment of cardiopulmonary capacity. Exercise testing or the measurement of the human body’s response to increased activity can be used to determine general cardiovascular fitness of an individual. It has also been utilized to assist in the determination of candidacy for successful utilization of a lower limb prosthesis among lower limb amputees [2]. Adequate physical fitness has also been identified as a correlate to increased function and successful ambulation with a prosthesis. However, these collective findings have never been compiled and assessed in a systematic way [3–6]. Further, there is no clear indication which modality of exercise testing is superior for use in the population with LEA and if exercise testing is best suited to determine general conditioning, prosthetic candidacy or ambulatory potential with a prosthesis. While guidelines for prosthetic candidacy as it pertains to cardiopulmonary function have been suggested in individual clinical trials, aggregation and synthesis of these findings and suggestions have not occurred.

**Fig. 1:**
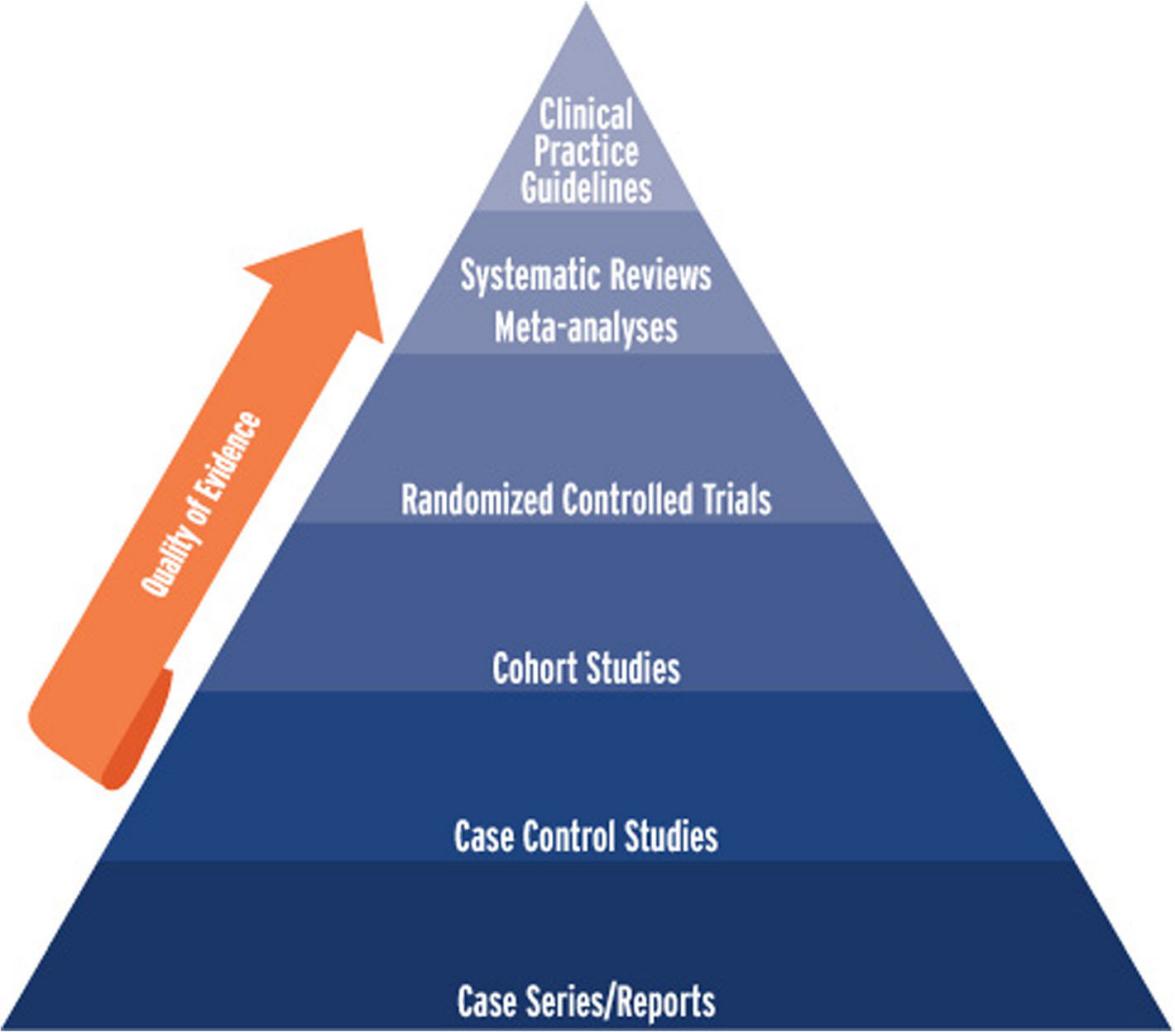
Heirarchy of research designs & levels of scientific evidence

These issues require consideration in their context of a healthcare climate moving toward evidence-based practice and the establishment of sound clinical practice recommendations, guidelines, and pathways [7]. These directives, to hold the most power and accuracy, must be based on the highest levels of research quality in systematic literature reviews and meta-analyses (Fig. 1).

Therefore, the purpose of this project was to perform a systematic literature review regarding the use of exercise testing in the population of patients with history of lower extremity amputation and use the synthesis of that review to establish clinical practice recommendation and present a clinical implementation pathway for future adoption.

## Methods

A multidisciplinary team developed the methodology to be used in this systematic literature review and guideline development. All procedures were in accordance with the PRISMA (Preferred Reporting Items for Systematic Reviews and Meta-Analyses) statement and the Clinical Practice Guideline Development Manual [7, 8]. The selected methodologies have been used in systematic reviews regarding prosthetic rehabilitation prior to this review as well as in the development of clinical practice guidelines and recommendations [9–13]. The multidisciplinary team consisted of a vascular surgeon, orthopedic surgeon, physical medicine and rehabilitation physician, research scientists, exercise physiologists, physical therapists, prosthetists, and epidemiologists from the private, Military, Veterans Affairs, and academic sectors. Many of the authors have experience in systematic literature reviews and creation of clinical practice recommendations and all have experience working directly with patients having a history of limb loss.

### Literature search

Searches of four electronic medical research databases including (1) PubMed, (2) Google Scholar, (3) Web of Science, and (4) the Cochrane Library were used to compile possible articles and was conducted in January 2017. The range of article publication dates included December 2001–December 2016. The searches were executed independently by two authors who have experience in performing literature database searches.

The search terms were developed by the multi-disciplinary team and used for all searches performed. The search term set includes the following, where the use of an asterisk (*) indicates the use of a MeSH term or analogous input:

amput* OR limb loss OR prosthe* AND function* OR capacity OR aerobic OR anaerobic OR cardio* OR vo2 maximum OR metabol* OR oxygen OR energy OR uptake OR consum* OR cost OR expend* OR fitness AND exercise OR stress OR ergomet* OR cycl* OR test* OR walk* OR ambulat* OR run*

The resultant collection of manuscripts was compiled and screened independently by the same two authors who completed the searches. The screeners eliminated duplicate articles between databases and articles which did not meet the following inclusion criteria:

- Human subjects
- Adult subjects (age 19 or older)
- Article written or translated to English
- Peer-reviewed
- Full-text available to authors
- Article included subjects with lower extremity amputation
- Article included results of an exercise testing modality

Article titles and abstracts were then reviewed and articles eliminated for relevance to the topic which was reporting the results of amputee subjects performing at least one exercise testing modality which was related to some other aspect of prosthetic rehabilitation. Full-text evaluations of the remaining articles were performed to determine final eligibility for the review. A further inclusion criteria for the clinical practice guideline (CPG) was direct applicability, meaning the articles utilizing solely able-bodied individuals as subjects were eliminated.

Included articles were aggregated and descriptive statistics were calculated for sociodemographic data as appropriate. Common outcome variables reported in exercise testing manuscripts were also identified and similarly analyzed. These variables were heart rate (HR), oxygen consumption (VO_2_), %VO_2max_, and maximum workload achieved during the testing protocol.

### Quality assessment

Manuscripts were assessed for quality using the critical appraisal tool outlined in the United Kingdom National Service Framework for Long-Term Conditions (UK NSF) [14]. This metric was developed by a UK Department of Health initiative in March 2005. This tool was selected as it has been used to develop clinical practice guidelines and is the preferred tool for the medical association of a developed, first-world country where no equivalent tool has been developed in the United States. The UK-NSF tool also allows for evaluation of a range of research types from case studies to meta-analyses, synthesis of empirical evidence statements (EESs), and grading of those statements for use in a clinical practice recommendation. The UK-NSF assesses design, applicability, and quality of the articles with quality being scored based on five questions:

1. Are the research questions/aims and design clearly stated?
2. Is the research design appropriate for the aims and objectives of the research?
3. Are the methods clearly described?
4. Is the data adequate to support the authors interpretations/inclusions?
5. Are the results generalizable?

These questions are each given a score according to the answer, where with 0 = no, 1 = in part, 2 = yes. These point values are then added for a score out of 10 where 7–10 is *high*, 4–6 is *medium*, and 0–3 is *low*, quality. Each article was assessed independently by two graders experienced in scoring articles with this tool and scores were agreed upon by a consensus of the multi-disciplinary team. Empirical evidence statements were then synthesized by the group and given research grades according to the criteria provided in the UK-NSF document [14]. Statements with Research Grades of A or B were incorporated into the resultant CPG.

## Results

**Fig. 2:**
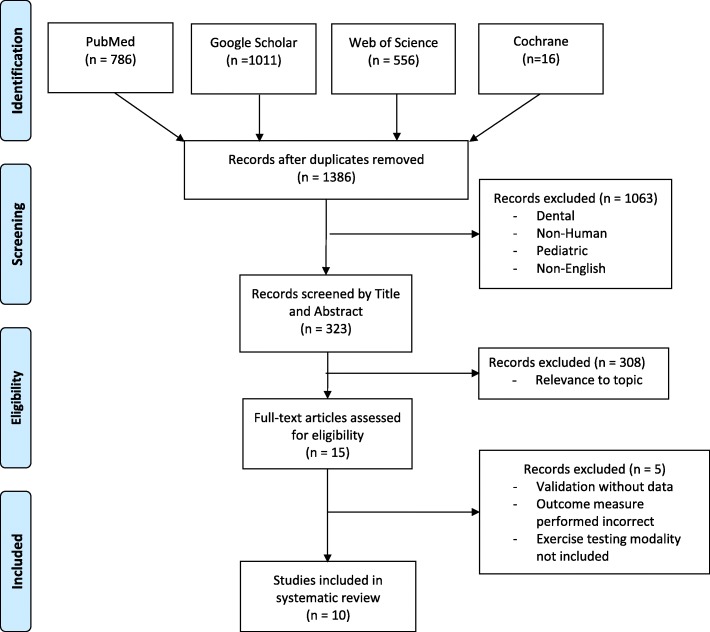
PRISMA flow diagram

Out of the 323 potentially relevant studies, 308 were excluded mostly due to lack of an exercise testing modality other than simple ambulation or those not related to an outcome variable associated with prosthetic rehabilitation. The remaining 15 full-text manuscripts were read and a further five eliminated due to being solely a validation study with no useable data, incorrect application of an outcome measure, or inclusion of only able-bodied individuals as the sole subjects. The PRISMA diagram is shown in Fig. 2. Of the remaining 10 studies, eight studies were determined to be high-quality, one medium-quality, and remaining study was rated as low-quality.

In terms of quality, five were prospective trials, four were retrospective cohort or mixed cohort designs, and one systematic literature review without data aggregation was included in this review. Seven of the 10 articles utilized captive samples from inpatient prosthetic rehabilitation programs in university or institutional medical systems with one study utilizing a convenience community sample. The remaining two studies did not report sample selection procedure. Half of the articles were written by a group of authors from a university hospital in Japan while two were written by one author in Slovenia and the remaining three articles originated from two groups in the Netherlands.

The sample includes 448 subjects included in the aggregation of data. Seventy-eight of these subjects were extracted from the included systematic review to include only those who had completed an exercise testing modality [15]. Attrition included 40 subjects for an overall attrition rate of 8.9%. An additional 39 able-bodied control subjects were studied and aggregated separately. Data from one subject was excluded as she was below 18 years of age. Demographic data from the sample is shown in Table 1.

**Table 1:** Subject sociodemographic data

Author	Journal	Year	n	age (years)	Weight (kg)	Etiology of Ampuation
Chin et al.	J Rehabil Res Dev	2012	7	67.7 ± 3.91	51.1 kg ± 9.7	tumor, infection
Chin et al.	Am J Phys Med Rehabil	2002	31 [18]	26.0 ± 5.7 [25.4 + 3.7]	X	X
Chin et al.	Am J Phys Med Rehabil	2006	49	67.5 ± 5.6 yrs	X	vascular, trauma, tumor, infection
Chin et al.	Prosthet Orthot Int	2002	17	67.4	X	vascular
Erjavec et al.	Disabil Rehabil	2014	101	69.4 (53–84)	X	vascular
Erjavec et al.	Eur J Phys Med Rehabil	2008	61	72.5	X	vascular
Hamamura, et al.	J Int Med Res	2009	64	67.3	X	Vascular, Non-vascular
Vestering, et al.	Int J Rehabil Res	2005	4	38.5	79.8 kg	Trauma, cancer, diabetes, neurofibromatosis
Wezenburg et al.	Ann Phys Med Rehabil	2012	36 [31]	62.3 [60.8 ± 5.9]	82.4 [81.1 kg ± 14.3]	traumatic, vascular
Van Velzen et al.	Disabi Rehabil	2006	78	70	X	Vascular
	Total		448	65.4 [44.5]	77.5	

Control subject data are presented in brackets []

Four exercise testing modalities were identified in the review; 1) single-leg ergometry (SLE), 2) upper extremity ergometry (UEE), 3) combined upper extremity/lower extremity ergometry (UEE/SLE), and 3) a rowing machine (RM). Of the six SLE tests identified, five followed a continuous testing protocol while one test followed an intermittent submaximal protocol in which the subject pedaled for 90 s and rested for 30 s between each metered effort increase. Of four UEE tests, two were continuous, one was intermittent in which the subject pedaled the UEE for 2 min with 1 min of rest between effort increases, and one followed an unreported protocol. The lone UEE/LE combined test, which was performed on a recumbent elliptical ergometer, was continuous. The protocol adopted on the RM was not reported. Study data is given in Table 2.

**Table 2:** Study data

Author	Journal	Year	Study Design	Amputation Level	Exercise Testing Modality	%VO_2max_	Maximum workload (W)	Attrition rate
Chin et al.	J Rehabil Res Dev	2012	Prospective Cohort	Hip Disarticulation	1-leg ergometer	57.2 ± 11.1%		0%
Chin et al.	Am J Phys Med Rehabil	2002	Prospective Cohort	Lower Extremity	1-leg ergometer	80.00%	67.6 + 20.2 W	0%
Chin et al.	Am J Phys Med Rehabil	2006	Prospective Cohort	Transfemoral/Hip Disarticulation	1-leg ergometer	64.4 ± 14.4%	X	0%
Chin et al.	Prosthet Orthot Int	2002	Retrospective Cohort	Transfemoral	1-leg ergometer	58.6 ± 7.6%	X	0%
Erjavec et al.	Disabil Rehabil	2014	Prospective Cohort	Unilateral Transfemoral	Upper Extremity	X	50 W	37%
Erjavec et al.	Eur J Phys Med Rehabil	2008	Prospective Cohort	Transfemoral	Upper Extremity	X	40 W	1%
Hamamura, et al.	J Int Med Res	2009	Retrospective Cohort	History of TFA or HD	1-leg ergometer	58.80%	X	X
Vestering, et al.	Int J Rehabil Res	2005	Case Series	Unilateral Lower Extremity	combined upper/lower extremity ergometer	63.69% (combined), 73.3% (UE)	95 W (combined), 106.7 W (UE)	20%
Wezenburg et al.	Ann Phys Med Rehabil	2012	Retrospective Cohort	Transtibial, transfemoral	1-leg cycle ergometer	X	132.0 W peak	3%
Van Velzen et al.	Disabi Rehabil	2006	Systematic Review	Lower Extremity	Rowing machine, UE ergometer	X	Level 2: 44 + 3 W, Level 3: 71 + 4 W	N/A

In terms of reported outcome measures, two articles reported HR, five on %VO_2max_, one on VO_2_ only, and five on maximum workload achieved during the testing protocol. Descriptive statistics were performed on %VO_2max_ and maximum achieved workload to determine measures of central tendency weighted by the number of subjects included in the applicable studies. %VO_2max_ is presented as a percent of predicted VO_2max_ calculated using the method described by Hansen et al. [16, 17] or as reported in the respective articles. Percent VO_2max_ was calculated post hoc in one study [4] in relation to the results of the age-matched, able-bodied controls completing the same protocol. Weighted means are given in Table 1.

Available data were synthesized into EESs and assigned research grades based on the guidelines established in the UK-NSF document [14]. Statements had to be supported by at least two articles to be synthesized. The review’s six synthesized statements are included in Table 3.

**Table 3:** ᅟ

Empirical Evidence Statements (EES)	Supporting Articles
The single-leg continuous maximal cycle ergometer test propelled by a sound limb is viable for evaluation of cardiorespiratory fitness using the percent achieved of a predicted VO_2max_ value (%VO_2max_) measured using direct spirometry in subjects with unilateral lower extremity limb loss.	3,4,5,6,19
The ability to sustain an exercise intensity of ≥50%VO_2max_ during a continuous maximal cycle ergometer test propelled by a single, sound limb is a strong predictor of the ability of the elderly subject with lower extremity limb loss proximal to the knee to successfully ambulate 100 m with a prosthesis.	5,6,19
The ability to sustain an exercise intensity of ≥60%VO_2max_ during a continuous maximal cycle ergometer test propelled by a single, sound limb is a predictor of the ability of an elderly, non-vascular subject with unilateral hip disarticulation to ambulate with a prosthesis.	3,5,19
The upper extremity intermittent submaximal cycle ergometer test is viable for evaluation of physical performance using achieved maximum workload (in W) of elderly subjects with lower extremity limb loss.	2,20,26
Achievement of 30 W on a submaximal intermittent upper extremity cycle ergometer test is a strong indicator of the ability of the elderly subject with history of transfemoral limb loss secondary to vascular etiology to successfully ambulate with a prosthesis.	2,15,20
Subjects with history of lower extremity amputation who do not achieve recommended levels of cardiorespiratory fitness or physical function in pre-prosthetic exercise testing should be prescribed a supervised physical rehabilitation program,preferably including ergometry with the sound lower extremity, and re-evaluated upon its completion. (E2)	6,20,26

All empirical evidence statements were supported by Grade A evidence. EES 6 was also designated the grade of E2 indicating synthesis by professional expert opinion.

**Fig. 3:**
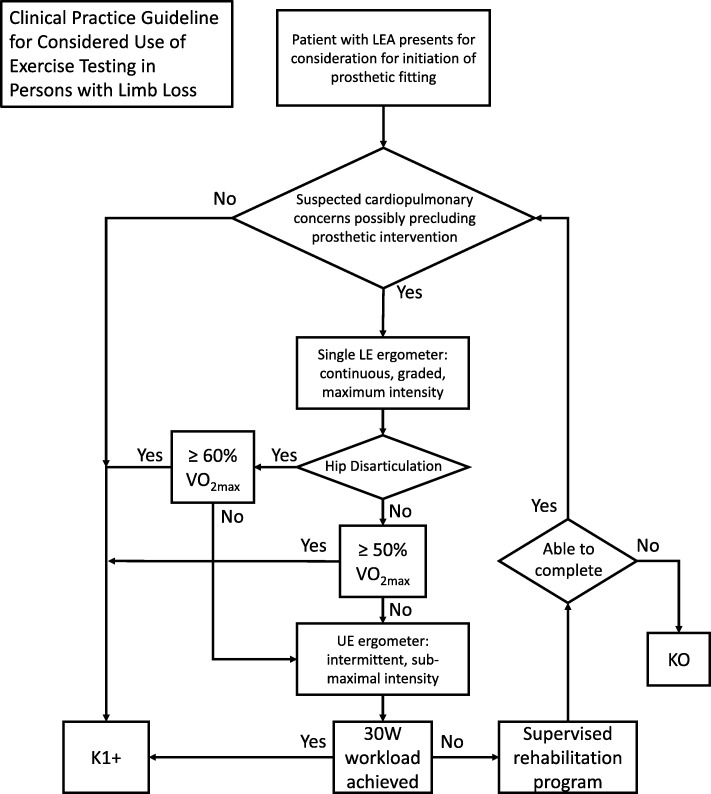
Clinical Practice Guideline (CPG) for considered use of exercise testing in persons with limb loss

The CPG developed from the findings of this review is outlined in the discussion and is presented in illustrated form in Fig. 3. The CPG depicts a pathway whereby a patient with limb loss suspected of cardiovascular compromise may benefit from results of exercise testing to clarify the extent of their conditioning or their candidacy for prosthetic ambulation. It is intended only for use when there is reasonable concern of cardiopulmonary compromise or impairment in a patient which would limit prosthetic use or function. The organization and synthesis of statements into the CPG were based on the expert opinion of the multidisciplinary team.

## Discussion

A systematic literature review regarding exercise testing in the population of individuals with history of lower extremity amputation was performed and articles were evaluated using the UK-NSF critical appraisal tool. This review served as the basis for development of CPG for the use of exercise testing in evaluation of prosthetic candidacy for those individuals with limb loss who are suspected to have cardiovascular compromise. Guidelines may prove useful when an individual with LEA presents for an initial of prosthetic fitting as well as a subsequent prosthesis. The CPG should not be applied to any case where reasonable concern for cardiopulmonary impairment or compromise are not present. All CPGs must also be based in evidence and efficient. Therefore, the aforementioned endpoints will be connected using highly-graded synthesized evidence statements supplemented with expert opinion from a consensus of the multi-disciplinary team only when necessary.

Although all synthesized EESs received the highest research grade provided by the framework, the use of SLE and %VO_2max_ calculation as an evaluation for cardiorespiratory fitness in subjects with LEA had the greatest number of evidentiary support articles. Viability of the SLE test was made by *Chin* et al. in 1997 [3] and since most of these articles were produced from the same group, the method for VO_2max_ prediction is the same between studies and is also commonly used [4–6, 16, 18, 19]. Two definitive statements can be made regarding correlation to successful prosthetic ambulation using this test. The first is that a subject’s ability to sustain ≥50%VO_2max_ is likely an indicator for successful prosthetic ambulation in the elderly subject with LEA proximal to the knee. Although the definition of elderly varies among the studies in this review many articles have found prosthetic function to decrease with age and, conversely, to increase with youth [2, 10, 20]. For this reason, it can be assumed, at least for the purposes of this synthesis, that achievement of a %VO_2max_ value or workload sustained by an elderly individual which would predict prosthetic ambulatory success would also indicate the likelihood of prosthetic ambulatory success to be achieved in younger subjects. Similarly, energy expenditure has been found to increase and function to decrease with more proximal amputation levels [21, 22]. Therefore, achievement of a minimum %VO_2max_ value or workload which would predict prosthetic ambulatory success of a subject with a proximal level of amputation would also indicate the likelihood of prosthetic ambulatory success in subjects with more distal amputation levels.

The second statement regarding SLE testing is the ability to sustain an exercise intensity ≥60%VO_2max_ by an older individual with unilateral hip disarticulation indicating anticipated ability to ambulate successfully with a prosthesis into the community ambulation level. A point of emphasis for both these statements regarding SLE is that the criteria for classification of successful ambulation was set at 100 m. This value is consistent with the work of Gailey et al. who correlated distance walked in the 6-min walk test to MFCL groups in their validation of the amputee mobility predictor [23]. This distance falls between the K0–1 group and K2 group in that study, meaning between household and community ambulators. This could indicate the 100 m distance as a beneficial outcome to further correlate measures against when developing criteria for determining of prosthetic candidacy.

One of the studies by Chin et al. [4] included in this review studied a sample of young adult subjects with limb loss and compared them to a control group of similarly-aged able-bodied controls. VO_2_ was found to be slightly lower in the subjects with limb loss and %VO_2max_ was calculated from the results of the control group post hoc for consistent comparison among included studies. Control subjects had higher absolute VO_2_ and sustained a higher maximum workload. This outcome trend was similar to other controlled trials regarding individuals with limb loss [24, 25]. After testing, subjects with LEA completed a 6-week cardiovascular training program with the SLE machine and were able to restore VO_2_ measurements and workload values to those of their able-bodied peers. While no study identified in this review demonstrated the effect of a rehabilitation program to improve cardiorespiratory capacity of a subject below the levels specified to indicate successful prosthetic use to surpass them, a similar positive outcome was also concluded by Erjavec et al. in a study using the UEE modality [20]. The multidisciplinary workgroup therefore suggests any patient unable to meet the recommended criteria of either UEE or SLE test should be given the option to complete an exercise program and re-enter the protocol before being disqualified from prosthetic intervention if desired.

Making recommendations for type of exercise regimen is beyond the scope of this review. However, it was previously noted that training on either UEE or SLE modality resulted in fitness gains. It is noted in Vestering et al. [26] that a combined UEE/SLE modality places a larger cardiopulmonary demand than does UEE alone, so any pre-prosthetic rehabilitation program should incorporate SLE or combined UEE/SLE elements as its basis. Further investigation in this area is needed.

The remaining modality in this review after SLE testing with enough evidence to be synthesized is the intermittent UEE test. The conclusions regarding this modality are based on maximum achieved workload in Watts sustained during the test. The work of Erjavec et al. supports the achieved workload of 30 W in the UEE test to correspond to successful prosthetic ambulation in the elderly transfemoral subject which should assume successful ambulation in the younger and also non-vascular subjects [26]. However, it should be noted that the literature reports there is greater cardiovascular and ventilatory strain (increased heart rate, blood pressure and respiratory rates) associated with upper extremity exercise compared to lower extremity at any given power output or % exercise intensity [27–29].

The final testing modality included in this review is the rowing machine which was one of the articles mentioned in the systematic review performed by Van Velzen et al. [15]. Although no causative relationship between achieved workload and aerobic capacity was concluded, it should be noted that 45 W sustained maximum in the rowing machine test was correlated to a cut-off point between those who met criteria of level 2 or level 3 prosthetic ambulator [15]. It was also found that subjects who achieved this wattage level were less likely to walk with a walker in favor of a higher-level assistive device or independently [15]. Further research is needed regarding the use of RM tests in the population of individuals with limb loss to be included in a proposed CPG at this time, although initial findings of the modality show promise.

### Limitations

A criticism of this study design may be that the systematic literature review without meta-analysis should not constitute the basis for a CPG. However, descriptive literature reviews have been used as the basis for clinical practice recommendations in the field of endoskeletal prosthetics as the literature base is currently limited compared to other healthcare fields [9, 11, 13]. Meta-analysis was considered in the early stages of this project, but the heterogeneity of outcome measures in terms of modality, intensity, and continuity limited the clinical meaningfulness of any results. It was also determined that the CPG, which is the primary goal of this project, would not benefit any further from performance of such an analysis except in perceived power.

An additional limitation of the review and resultant CPG could be the lack of functional level stratification beyond basic candidacy at the K1 level. It should be noted the intended purpose of this pathway is only for those individuals with limb loss and suspected comorbid cardiovascular compromise which may limit prosthetic use who are seeking evaluation for an initial prosthesis or subsequent prosthesis when other factors including present fitness level may render completion of a walking test unsafe or impractical. Testing of this type is not commonly used in the population with LEA and, in practice, most amputee subjects would more commonly be able to perform a walking test or battery such as the amputee mobility predictor. Therefore, it is the lower-functioning patient with multiple comorbidities in the early stages of rehabilitation who would benefit most from an alternative set of criteria for determination of candidacy and functional classification when access is being limited. A larger review seeking values featured in the results of this review would allow for both the cited limitations of the study to be addressed in the future [30].

It should be noted that none of the studies identified in this review investigated the effect of an exercise program to increase cardiovascular capacity from below to above recommended values indicating the predicted success of ambulation with a prosthesis. Therefore, while the recommendation of such exercise program may be considered medically and clinically reasonable it has not yet been directly studied. It is for this reason that the final EES was also given an evidence grade of E2 indicating support by professional expert opinion.

Lastly, none of the articles included in this review included indirect estimation of VO_2_ based on HR. Although direct spirometry is the most accurate mode of measuring gas exchange, this equipment is expensive and not often available in general rehabilitation centers which could potentially limit access and delay time to prosthetic fitting. However, a comparison of indirect estimation of VO_2_ to direct measurement in the population with LEA using the Fick equation or similar validated method could greatly increase access to testing as many physical therapy clinics have at least one of the modality types mentioned in this review [31, 32]. The dependence of this review and CPG on direct spirometry limits the clinical applicability at this time. Further research correlating indirect estimation of VO_2_ and direct spirometry in the population with LEA is needed.

## Conclusions

A systematic review of literature regarding the use of exercise testing in treatment of the population with limb loss was completed. Data was synthesized into six empirical evidence statements and founded the basis for a CPG developed by a multi-disciplinary research team. Both continuous maximal lower extremity ergometer and intermittent sub-maximal upper extremity ergometer protocols were found to be viable methods of evaluating cardiorespiratory fitness and function in persons with limb loss. Additional modalities included combined upper and lower extremity ergometers and rowing machine protocols. VO_2max_ reported as a percent of a normalized predicted value was the most commonly reported outcome and 50%VO_2max_ was repeatedly found to be a reliable threshold for successful ambulation with a prosthesis. Achievement of a sustained workload of 30 W on the upper extremity ergometer protocol was also found to be a reliable correlate to successful prosthetic ambulation. The development and presentation of this clinical practice guideline may provide an alternative method of functional level classification of patients with history of limb loss and its adoption should only be used to increase access to prosthetic interventions and rehabilitation for these individuals.

## Additional file

Additional file 1: Dear Physician Letter.ᅟ(PDF 469 kb)

## Abbreviations

CPG: Clinical practice guideline
EES: Empirical evidence statement
HR: Heart rate
LEA: Lower extremity amputation
SLE: Single-leg ergometry
UEE: Upper extremity ergometry
UK-NSF: United Kingdom National Service Framework for Long-Term Conditions
VO_2_: Oxygen consumption
