# Supplementary material for: The role of exercise testing in predicting successful ambulation with a lower extremity prosthesis: a systematic literature review and clinical practice guideline
Source: J Neuroeng Rehabil. 2018 Sep 5;15(Suppl 1):64. doi: 10.1186/s12984-018-0401-z (PMC6156901; doi:10.1186/s12984-018-0401-z)
Supplement: Supplementary file 1 — Dear Physician Letter.ᅟ(PDF 469 kb) [file 12984_2018_401_MOESM1_ESM.pdf]

noridian
Healthcare Solutions

August 2011

Documentation of Artificial Limbs

Dear Physician,

The Durable Medical Equipment Medical Administrative Contractors (DME MAC) have jurisdiction for processing claims from prosthetists for artificial limbs. In the event of an audit, the Medicare contractor may request medical records to demonstrate that the prosthetic arm or leg was reasonable and necessary. Since the prosthetist is a supplier, the prosthetist's records must be corroborated by the information in your patient's medical record. It is the treating physician's records, not the prosthetist's, which are used to justify payment.

The patient's functional capabilities are crucial to establishing the medical necessity for a prosthetic device. Many prosthetic components are restricted to specific functional levels; therefore, it is critical that physicians thoroughly document the functional capabilities of their patients, both before and after amputation. Clinical assessments of a patient's rehabilitation potential must be based on the following classification levels:

Level 0: Does not have the ability or potential to ambulate or transfer safely with or without assistance and a prosthesis does not enhance their quality of life or mobility.

Level 1: Has the ability or potential to use a prosthesis for transfers or ambulation on level surfaces at fixed cadence. Typical of the limited and unlimited household ambulator.

Level 2: Has the ability or potential for ambulation with the ability to traverse low level environmental barriers such as curbs, stairs or uneven surfaces. Typical of the limited community ambulator.

Level 3: Has the ability or potential for ambulation with variable cadence. Typical of the community ambulator who has the ability to traverse most environmental barriers and may have vocational, therapeutic, or exercise activity that demands prosthetic utilization beyond simple locomotion.

Level 4: Has the ability or potential for prosthetic ambulation that exceeds basic ambulation skills, exhibiting high impact, stress, or energy levels. Typical of the prosthetic demands of the child, active adult, or athlete.

The records must document the patient's current functional capabilities and his/her expected functional potential, including an explanation for the difference. Note that it is recognized, within the functional classification hierarchy, that bilateral amputees often cannot be strictly bound by functional level classifications.

CPT codes, descriptors and other data are copyright 2015 American Medical Association (or such other date of publication of CPT). All Rights Reserved. Applicable FARS/DFARS apply. This article applies to all NHS administered states unless otherwise noted in the article

The physician's assessment of a patient's physical and cognitive capabilities typically includes:

- History of the present condition(s) and past medical history that is relevant to functional deficits
- Symptoms limiting ambulation or dexterity
- Diagnoses causing these symptoms
- Other co-morbidities relating to ambulatory problems or impacting the use of a new prosthesis
- What ambulatory assistance (cane, walker, wheelchair, caregiver) is currently used (either in addition to the prosthesis or prior to amputation)
- Description of activities of daily living and how impacted by deficit(s)
- Physical examination that is relevant to functional deficits
- Weight and height, including any recent weight loss/gain
- Cardiopulmonary examination
- Musculoskeletal examination
    - Arm and leg strength and range of motion
- Neurological examination
    - Gait
    - Balance and coordination

The assessment points above are not all-inclusive and physicians should tailor their history and examination to the individual patient's condition, clearly describing the pre and post-amputation capabilities of the patient. The history should paint a picture of your patient's functional abilities and limitations on a typical day. It should contain as much objective data as possible. The physical examination should be focused on the body systems that are responsible for the patient's ambulatory or upper extremity difficulties or impact on the patient's functional ability.

Note that when physicians are unable to provide the requested documentation to the supplier, the suppliers receive denials for the items billed which could result in your patient being financially responsible for all or part of the charges for the items/service received. If a supplier contacts your office to request additional clinical documentation, please partner with the supplier to establish what clinical records are needed to support that the service/item you ordered is medically necessary.

Section 1842(p)(4) of the Social Security Act mandates that:

[I]n case of an item or service... ordered by a physician or a practitioner... but furnished by another entity, if the Secretary (or fiscal agent of the Secretary) requires the entity furnishing the item or service to provide diagnostic or other medical information in order for payment to be made to the entity, the physician or practitioner shall provide that information to the entity at the time that the item or service is ordered by the physician or practitioner.

CPT codes, descriptors and other data are copyright 2015 American Medical Association (or such other date of publication of CPT). All Rights Reserved. Applicable FARS/DFARS apply. This article applies to all NHS administered states unless otherwise noted in the article

Providing medical records to the supplier is not a violation of the HIPAA Privacy Rule. Thank you for your cooperation in future documentation requests.

Wilfred Mamuya, MD, PhD
Medical Director, DME MAC, Jurisdiction A
Noridian Healthcare Solutions

Robert D. Hoover, Jr., MD, MPH, FACP
Medical Director, DME MAC, Jurisdiction C
CGS Administrators, LLC

Stacey V. Brennan, MD, FAAFP
Medical Director, DME MAC, Jurisdiction B
CGS Administrators, LLC

Peter J. Gurk, MD, CPE, CHCQM
Medical Director, DME MAC, Jurisdiction D
Noridian Healthcare Solutions

CPT codes, descriptors and other data are copyright 2015 American Medical Association (or such other date of publication of CPT). All Rights Reserved. Applicable FARS/DFARS apply. This article applies to all NHS administered states unless otherwise noted in the article

For any item to be covered by Medicare, it must: 1) be eligible for a defined Medicare benefit category, 2) be reasonable and necessary for the diagnosis or treatment of illness or injury or to improve the functioning of a malformed body member, and 3) meet all other applicable Medicare statutory and regulatory requirements.

The purpose of a Local Coverage Determination (LCD) is to provide information regarding "reasonable and necessary" criteria based on Social Security Act § 1862(a)(1)(A) provisions.

In addition to the "reasonable and necessary" criteria contained in this LCD there are other payment rules, which are discussed in the following documents, that must also be met prior to Medicare reimbursement:

- The LCD-related Standard Documentation Requirements Article, located at the bottom of this policy under the Related Local Coverage Documents section.
- The LCD-related Policy Article, located at the bottom of this policy under the Related Local Coverage Documents section.
- Refer to the Supplier Manual for additional information on documentation requirements.
- Refer to the DME MAC web sites for additional bulletin articles and other publications related to this LCD.

For the items addressed in this LCD, the "reasonable and necessary" criteria, based on Social Security Act § 1862(a)(1)(A) provisions, are defined by the following coverage indications, limitations and/or medical necessity.

A lower limb prosthesis is covered when the beneficiary:

1. Will reach or maintain a defined functional state within a reasonable period of time; and
2. Is motivated to ambulate.

FUNCTIONAL LEVELS:

A determination of the medical necessity for certain components/additions to the prosthesis is based on the beneficiary's potential functional abilities. Potential functional ability is based on the reasonable expectations of the prosthetist, and treating physician, considering factors including, but not limited to:

The beneficiary's past history (including prior prosthetic use if applicable); and

The beneficiary's current condition including the status of the residual limb and the nature of other medical problems; and

The beneficiary's desire to ambulate.

Clinical assessments of beneficiary rehabilitation potential must be based on the following classification levels:

Level 0: Does not have the ability or potential to ambulate or transfer safely with or without assistance and a prosthesis does not enhance their quality of life or mobility.

Level 1: Has the ability or potential to use a prosthesis for transfers or ambulation on level surfaces at fixed cadence. Typical of the limited and unlimited household ambulator.

Level 2: Has the ability or potential for ambulation with the ability to traverse low level environmental barriers such as curbs, stairs or uneven surfaces. Typical of the limited community ambulator.

Level 3: Has the ability or potential for ambulation with variable cadence. Typical of the community ambulator who has the ability to traverse most environmental barriers and may have vocational, therapeutic, or exercise activity that demands prosthetic utilization beyond simple locomotion.

Level 4: Has the ability or potential for prosthetic ambulation that exceeds basic ambulation skills, exhibiting high impact, stress, or energy levels. Typical of the prosthetic demands of the child, active adult, or athlete.
